# Molecular Mechanisms and Pathophysiological Significance of Eryptosis

**DOI:** 10.3390/ijms24065079

**Published:** 2023-03-07

**Authors:** Sumiah A. Alghareeb, Mohammad A. Alfhili, Sabiha Fatima

**Affiliations:** Chair of Medical and Molecular Genetics Research, Department of Clinical Laboratory Sciences, College of Applied Medical Sciences, King Saud University, Riyadh 12372, Saudi Arabia

**Keywords:** eryptosis, calcium, oxidative stress, anemia, anti-eryptotic compounds

## Abstract

Despite lacking the central apoptotic machinery, senescent or damaged RBCs can undergo an unusual apoptosis-like cell death, termed eryptosis. This premature death can be caused by, or a symptom of, a wide range of diseases. However, various adverse conditions, xenobiotics, and endogenous mediators have also been recognized as triggers and inhibitors of eryptosis. Eukaryotic RBCs are unique among their cell membrane distribution of phospholipids. The change in the RBC membrane composition of the outer leaflet occurs in a variety of diseases, including sickle cell disease, renal diseases, leukemia, Parkinson’s disease, and diabetes. Eryptotic erythrocytes exhibit various morphological alterations such as shrinkage, swelling, and increased granulation. Biochemical changes include cytosolic Ca^2+^ increase, oxidative stress, stimulation of caspases, metabolic exhaustion, and ceramide accumulation. Eryptosis is an effective mechanism for the elimination of dysfunctional erythrocytes due to senescence, infection, or injury to prevent hemolysis. Nevertheless, excessive eryptosis is associated with multiple pathologies, most notably anemia, abnormal microcirculation, and prothrombotic risk; all of which contribute to the pathogenesis of several diseases. In this review, we provide an overview of the molecular mechanisms, physiological and pathophysiological relevance of eryptosis, as well as the potential role of natural and synthetic compounds in modulating RBC survival and death.

## 1. Introduction

Erythrocytes or red blood cells (RBCs) transport gases (O_2_ and CO_2_) between tissues and lungs, and maintain acid/base equilibrium in the body. Being the most abundant cell type in the body, they serve as an important health indicator. Analysis of the erythrocyte membrane phospholipid organization of the outer leaflet is crucial to understand the role of cell membrane biology in health and disease [1]. Although, the erythrocytes lack important organelles, such as mitochondria and a nucleus, which are required in the execution of apoptosis, damaged RBCs can undergo programmed cell death-like apoptosis, called eryptosis [2,3].

Injury of the cells leading to the death of the erythrocytes can be triggered by various exogenous factors, such as xenobiotics, toxins, and heavy metal exposure, along with the administration of antibiotics [4,5,6]. Moreover, several physiological dysfunctions, such as oxidative stress, osmotic shock, energy depletion, hyperosmolarity, and release of prostaglandin E2 (PGE2), induce a rapid self-destruction process in mature erythrocytes [7,8].

The characteristics of eryptosis involves dysfunctional ion exchange, cell shrinkage, ceramide accumulation, cell membrane vesiculation due to cytosolic calcium overload, and membrane phospholipid scrambling with alteration of the cell membrane asymmetry and phosphatidylserine (PS) exposure at the cell surface [9,10]. Ca^2+^ influx opens Ca^2+^-sensitive K^+^ channels, or Gardos channels, leading to subsequent loss of KCl, hyperpolarization of the membrane, loss of water, and cell shrinkage [11,12]. Further, Ca^2+^ influx also stimulates the formation of ceramide, which causes disruption of cell membrane asymmetry [12,13]. Plasma membrane asymmetry plays a critical role in the function of membrane proteins and in the interactions with the cytoskeleton, influencing the mechanical properties of the cell and performing a key role in the integrity of the RBC [14]. An increased number of erythrocytes with membrane dysfunction can lead to seriously detrimental health conditions, such as anemia, microcirculation dysfunction, and thrombogenic activation [8]. It contributes to the pathogenesis of several clinical conditions, such as anemia, chronic kidney disease, Wilson’s disease, liver failure, diabetes, hypertension, heart failure, thrombosis, obesity, metabolic syndrome, arthritis, and lupus, which not only exacerbates fatigue but is also correlated with lower survival rates [15,16]. Moreover, diabetes-induced renal failure, hypertension, and sepsis are all associated with impairments in erythrocyte deformability [17].

From a physiological point of view, eryptosis is a protective mechanism mostly similar to the apoptosis of nucleated cells, to remove defective erythrocytes. It is an important process as it enables a form of erythrocyte cell death other than hemolysis, which results in rupture of the membranes with release of damaged RBC contents and consequent inflammation [18]. Thus, a balance between eryptotic and anti-eryptotic mechanisms is important to maintain a normal erythrocyte count to prevent blood associated irregularities. However, excessive eryptosis without accompanying erythropoiesis and enduring an increase of reticulocytes initiates phagocytosis of RBCs and compromises microcirculation by loss of circulating erythrocytes [16]. The process of eryptosis regulation is complex, implicating a multitude of cellular machinery, and there are various triggers and inhibitors. Several kinases, including cyclin-dependent kinase 4, p38 mitogen-activated kinase, casein kinase 1α (CK1α), mitogen- and stress-activated kinase MSK1/2, and Janus-activated kinase JAK3, have been shown to participate in the stimulation or inhibition of eryptosis [10,19]. Many food-derived phytochemicals and natural compounds, including phenolic compounds and alkaloids, with antioxidant and anti-inflammatory properties have been reported to prevent eryptosis-driven cell death and associated clinical conditions [20].

## 2. Structural Stability of RBCs

In RBCs, the cytoskeleton and plasma membrane are tightly coupled to form a basic and intricate structure known as the membrane skeleton. This is crucial for the structure and deformability of RBCs. Due to maintenance of the membrane structural integrity, RBCs remain flexible and are able to survive in the circulation [21].

In mature RBCs, the membrane is formed of a very particular lipid bilayer and interacts with other membrane proteins through protein–protein interactions. All cells have a double-layered surface called a cell membrane, which is composed of phospholipids, glycolipids, and cholesterol. While cholesterol is normally uniformly distributed across the two leaflets, the four major phospholipids are organized in an asymmetrical fashion [21].

The structural integrity of the RBC depends on the phospholipids, namely the PS and phosphoinositides, being arranged in an asymmetrical fashion in the inner leaflet [22]. Disruption of this lipid asymmetry results in PS appearing on the outer leaflet, which may contribute to the rapid mortality of RBCs [5,23]. Markers, such as cytosolic Ca^2+^ and increased hemoglobin, are present in the cytosol. Phospholipid bilayers have a role in lipid homeostasis through enzyme activities, membrane transport, and signal transduction. Polar hydrophilic heads and non-polar hydrophobic hydrocarbon tails give the numerous different molecular species of phospholipids their distinctive identities. Sphingomyelin is composed of sphingosine instead of glycerophospholipids [24,25].

To facilitate physiological activities, such as signal transduction, the membrane forms specialized portions called rafts, which are composed of clumps of lipids and proteins. Cholesterol in the membrane is sequestered between two lipid bilayers, where it cannot be sterilized. In lipid rafts, which are rich in cholesterol and sphingolipids, Flotillins, stomatin, G-proteins, and adrenergic receptors are found [26]. The lipid bilayer is composed of two types of phospholipids: choline-phospholipids (phosphatidylcholine [PC]) and amino-phospholipids (phosphatidylserine [PS] and phosphatidylethanolamine [PE]). Ceramide is the hydrophobic backbone and the most frequent and widespread type of the sphingolipid class [24,27].

**Figure 1:**
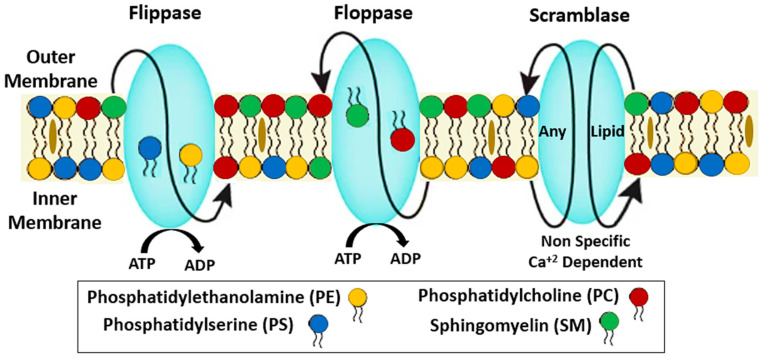
Schematic representation of lipid transbilayer transporters in the generation and maintenance of plasma membrane asymmetry. Flippases mediate ATP-dependent translocation of phosphatidylserine (PS) and phosphatidylethanolamine (PE) from the extracellular to the inner cytosolic side. Floppases catalyze the ATP-dependent translocation of phosphatidylcholine (PC), sphingomyelin (SM), and cholesterol to the outer leaflet. Scramblases promote Ca^2+^-activated nonspecific bidirectional movement.

There are three different protein classes that help in the transmembrane passage and lipid structural organization in the RBC membrane (Figure 1). Floppase regulates the movement of PS, SL, and cholesterol in the opposite direction, from the inner to the outside leaflet, where choline-containing phospholipids are stored (against concentration gradients). The aminophospholipidtranslocase, or flippase, moves phospholipids containing amino acids from one leaflet to the other [28]. Scramblase triggers the energy-free, concentration-dependent transport of phospholipids across the RBC membrane. Activation of phospholipid scramblase has been connected to the loss and disruption of the membrane’s asymmetry of phospholipids, which is a crucial mechanism for maintaining stable RBC lipid levels [25,28].

As a result of exchanging phospholipids from the outer monolayer for ATP, magnesium-ATP-dependent flippase can keep phospholipids in the correct proportions. The asymmetry of phospholipids is broken down and PS is exposed due to an action that scrambles phospholipids. Removal of PS from the inner leaflet of RBCs requires an increase in intracellular Ca^2+^, which in turn inhibits the flippase and activates the scramblase [29]. Energy-independent flippase catalyzes the synthesis of a variety of glycoconjugates, including glycosphingolipids, N-glycoproteins, and GPI-anchored proteins [30]. They contribute to the rapid balancing of molecules of ubiquitous phospholipids between the two membranes. Scramblase is nonselective in its facilitation of the lipid switch between the inner and outer leaflets.

Lipids are transported from one monolayer to another by scramblase, with the direction of transport determined only by the concentration gradient. Band 3, one of the red blood cell’s structural proteins, plays a crucial role in anion transport across the RBC membrane and cytoskeleton organization when calcium is present. Band 3 is a multi-spanning, transmembrane protein or ion channel that anchors the lipid bilayer to spectrin. Its cytoplasmic domain interacts with ankyrin, which is connected with spectrin [5].

The RBC membrane contains nearly 850 minor and 20 major proteins that serve as a cytoskeleton. The cell membrane skeleton is a pseudohexagonal meshwork of spectrin, actin and its associated proteins (tropomyosin, tropomodulin, adducin, and dematin), protein 4.1R, and ankyrin. The function of these proteins is to maintain the stability of the membrane, the shape and flexibility of the cell, and to limit diffusion of integral proteins within the bilayer [31]. During oxidative stress, the sulfhydryl side chains of two cysteine residues react to form a disulfide bond that causes cross linking and aggregation of membrane skeletal proteins [32].

## 3. Biochemical and Molecular Mechanisms of Eryptosis

**Figure 2:**
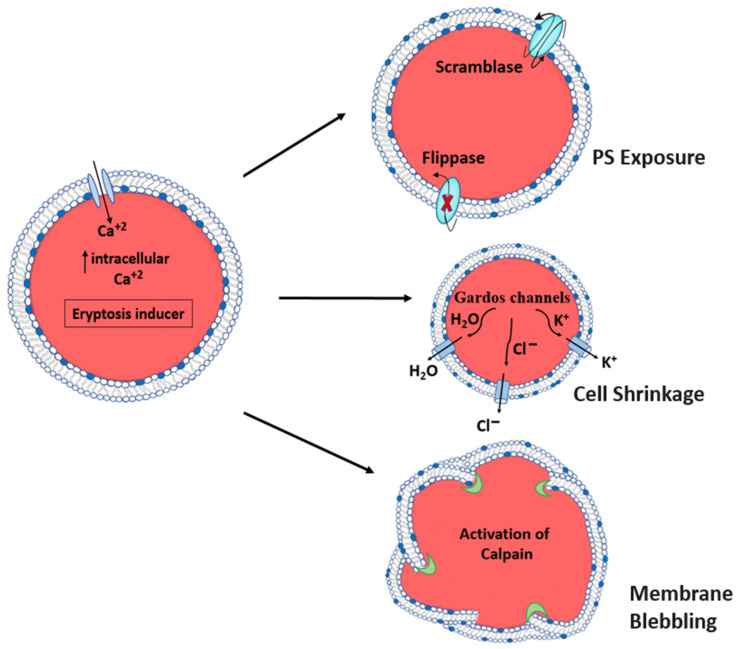
Mechanisms of Eryptosis. A key mechanism of eryptosis involves an increased intracellular Ca^2+^ concentration due to the activation of Ca^2+^-permeable cation channels facilitating the inflow of extracellular Ca^2+^ into the erythrocyte. (1) Increased intracellular Ca^2+^ consequently causes the activation of scramblases and inactivation of flippases, which results in exposure of phosphatidylserine (PS) at the cell surface. (2) Increased cellular Ca^2+^ concentration activates Ca^2+^-sensitive potassium channels, the Gardos channels. Activation of Gardos channels results in loss of water and KCl, which causes shrinking of the erythrocytes. (3) The Ca^2+^-activated cysteine endopeptidase calpain leads to degradation of the cytoskeleton, which in turn causes membrane blebbing.

Erythrocytes, under some conditions, incur a kind of cell death termed eryptosis before their full lifespan has been attained [33]. Many clinically relevant conditions and their treatments, such as oxidative stress, hyperosmolarity, heavy metal exposure, energy depletion, xenobiotics, and antibiotics, can trigger this kind of cell death [34]. Erythrocytes are continuously shuttled through high-pressure environments. The erythrocyte may be subjected to oxidative stress in the lungs or osmotic shock in the kidneys. Figure 2 shows the changes that occurs in the erythrocyte membrane, such as membrane blebbing, cell shrinkage, and phosphatidylserine exposure, all of which are features shared by apoptosis and eryptosis [35]. Osmotic shock and oxidative stress raise cytosolic calcium (Ca^2+^), triggering eryptosis [36]. PGE_2_ induces cell membrane vesiculation, enabling Ca^2+^ ions to enter erythrocytes. Ca^2+^ activates Gardos channels, causing the erythrocyte to leak KCl and water. Erythrocytes lose KCl and water during Ca^2+^-sensitive K^+^ channel-induced eryptosis [36]. PGE_2_ increases Ca^2+^ ion levels and exposes cell membrane PS when Cl^−^ ions are removed [36]. Cell membrane phospholipid scrambling exposes PS. After the erythrocyte’s PS is exposed, circulating macrophages with PS receptors identify and engulf it, removing it from the circulation [37,38].

During this process, the activation of the cysteine endopeptidase calpain degrades cytoskeleton proteins, such as ankyrin R complex, resulting in cell membrane blebbing and increased erythrocyte adhesiveness [20]. This phospholipid scrambling of the membrane causes shifting of PS from the inner to the outer cell membrane and disruption of cell membrane asymmetry [12,13]. Plasma membrane asymmetry plays a critical role in the function of membrane proteins and in the interactions with the cytoskeleton, influencing the mechanical properties of the cell [39]. Asymmetric arrangement of plasma membrane phospholipids, especially the organization of phosphoinositides and PS to the inner leaflet, performs a key role in the integrity of the RBC [14]. By facilitating enzymatic reactions participating in membrane transport and signal transduction pathways, the phospholipid bilayer plays a key role in lipid homeostasis [5]. The lipid-associated changes in membrane structure and properties change the entire signal transduction pathway, which may affect the cytoskeleton and plays major role in the premature destruction of RBCs [40]. The exposed PS on the erythrocyte cell membrane is recognized by circulating macrophages with specific phosphatidylserine receptors, which engulf and removal the erythrocyte from the circulation [39,41,42].

Additionally, cell death triggers the release of platelet activating factor (PAF). Sphingomyelinase, either endogenous to the erythrocyte or exogenous, disrupts sphingomyelin, releasing ceramide and contributing to the regulation of inflammation. Ceramide, when secreted into the plasma, elevates the amount of Ca^2+^-sensitive K^+^ channels present [34]. Because osmotic stress triggers the release of PAF via the activation of phospholipase, the presence of ceramide on the cell membrane causes PAF to generate a scrambled sarcolemma, resulting in the exposure of phosphatidylserine on the erythrocyte membrane. Ceramide’s possible role in inducing transbilayer lipid transport may explain this action [34].

Signaling molecules related to energy shortage further enhances eryptosis. Janus-activated kinase 3 (JAK3) is a transcription factor that phosphorylates tyrosine 980 (Tyr 980), an important tyrosine regulatory site [8,43]. When JAK3 is activated in response to energy deficiency, the cell membrane becomes scrambled. Additionally, casein kinase 1 (CK1) has been pharmacologically linked in the rise of Ca^2+^ ions and subsequent promotion of eryptosis in erythrocytes following energy deprivation or oxidative stress. When CK1 is activated by a pharmacological stimulus, it opens cation channels, allowing Ca^2+^ to enter the erythrocyte [8,43].

## 4. Physiological Roles of Eryptosis

Eryptosis is a physiological defense mechanism that shortens the life of erythrocytes and removes them from circulation after they have been damaged by injury or certain clinical conditions. Increased sensitivity of erythrocytes to hyperosmolarity, oxidative stress, and energy depletion occurs in genetic disorders, including sickle cell anemia, glucose-6-phosphate dehydrogenase deficiency, and thalassemia, shortening the erythrocyte lifespan and facilitating the elimination of the defective erythrocyte [43]. The distinctive rise of cytosolic Ca^2+^ ions that occurs during natural erythrocyte aging provides insight into the mechanism of eryptosis. Senescence is the process by which old erythrocytes are removed from the circulation because they are no longer able to withstand the stresses of circulation, such as oxidative stress, hyperosmolarity, and energy depletion [34] The process of eryptosis occurs physiologically to control and eliminate senescent and dysfunctional cells [44].

It is well established that hemolysis of damaged erythrocytes results in the release of the erythrocyte’s contents into the bloodstream, including hemoglobin, which can result in renal failure [43,45]. In addition, the accumulated heme and hemoglobin rapidly react with nitric oxide (NO), which reduces its availability in the circulation. The reduction in NO will result in vasoconstriction, upregulation of adhesion molecule expression, and endothelial activation [46]. The adhesion molecule, a pro-inflammatory ligand of innate immune receptors by activating the release of pro-inflammatory cytokines and chemokines, initiates the inflammatory process [47]. By providing another form of erythrocyte cell death, eryptosis removes the defective erythrocytes prior to hemolysis and thus prevents the complications associated with it. A balance between anti-eryptotic and eryptotic mechanisms is crucial to maintain a normal erythrocyte count to prevent blood irregularities [8].

Increased eryptosis is beneficial in the case of malaria since it limits the growth of the parasites in the erythrocytes. Infected erythrocytes undergo increased oxidative stress, which in turn causes Ca^2+^ ions to enter the erythrocyte via cation channels, setting off eryptosis, and eventually clearing the bloodstream of the infected cells [41]. To keep the erythrocyte count in the blood normal and to prevent anomalies, an equilibrium between the eryptotic and anti-eryptotic systems is essential. The proper quantity of erythrocytes in circulation relies on a balance between the eryptosis and hematopoiesis processes [43]. Any deregulation of these two regulatory processes may lead to the alteration in the number of circulating erythrocytes, affecting tissue oxygenation.

Fetuses and newborns have a unique kind of hemoglobin, fetal hemoglobin (HbF). HbF has a high oxygen affinity that favors effective oxygen transport in the low oxygen intrauterine environment [2]. However, after birth, for effective oxygen transport, replacement of HbF with adult hemoglobin is functionally essential. A newborn’s erythrocytes are resistant to various triggers of eryptosis, but they are highly susceptible to eryptosis following oxidative stress [48]. Due to its sensitivity to oxygen, HbF is eliminated from the newborn’s circulation once they have been exposed to inspired oxygen after delivery [2].

## 5. Modulation of Eryptosis

**Figure 3:**
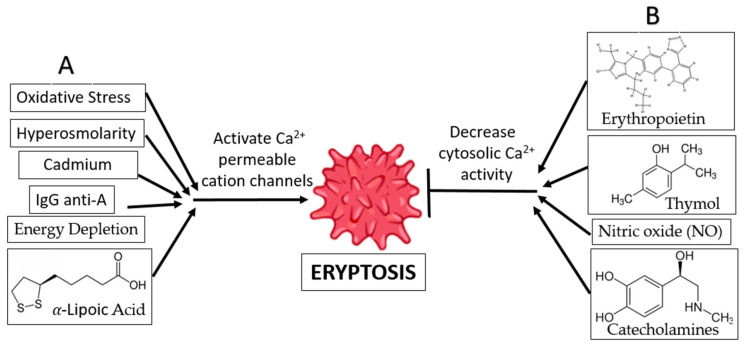
Modulation of Eryptosis. (**A**): Adverse conditions, as well as some compounds, can trigger eryptosis by increasing intracellular Ca^2+^ levels. (**B**): Many compounds can prevent eryptosis by decreasing the cytosolic Ca^2+^ activity.

As depicted in Figure 3, Premature death of RBCs can be triggered by several contributors, such as oxidative stress, energy depletion, xenobiotics, endogenous mediators, and adverse culture conditions [6,8,43,49].

(a) Oxidative Stress and Hyperosmolarity: Oxidative stress and hyperosmolarity activate Cl^−^ and Ca^2+^-permeable cation channels, as well as cysteinyl, aspartyl, and proteases [38]. Eryptosis is induced by a rise in intracellular Ca^2+^ levels, which is the result of PGE2 production in the absence of Cl^−^ ions. Furthermore, oxidative stress activates erythrocyte-produced caspases, resulting in enhanced PS exposure and recognition of the erythrocyte by circulating macrophages. The existence of hyperosmolarity does not require the activation of caspase [38].
(b) Energy Depletion: Inadequate glutathione (GSH) replenishment during calorie restriction has been associated with decreases in erythrocyte antioxidant activity. Energy deprivation also activates Ca^2+^-permeable cation channels in erythrocyte cell membranes, which in turn triggers eryptosis and the production of cyclooxygenase-2 (COX2) and (PGE2) [38]. Energy deprivation may also impact the phosphorylation of membrane proteins and the activity of the protein kinase C, resulting in the release of PS and the subsequent shrinking of the cell. Direct activation of eryptosis and an increase in intracellular Ca^2+^ ion concentration are the results of PKC activation [38].
(c) Hyperthermia: When subjected to hyperthermia, erythrocytes are incapable of upregulating the production of protective proteins, and increased Ca^2+^ entry results in uniform cell suicide. The increase in cytosolic Ca^2+^ activity affects the architecture of the cytoskeleton through a decrease in erythrocyte volume, membrane scrambling, and cell shrinkage, resulting in membrane blebbing, another characteristic of eryptosis [50].
(d) α-Lipoic Acid: Ca^2+^-sensitive K^+^ channels are activated in response to exposure to α-lipoic acid, leading to erythrocyte shrinking. This phenomenon may be partially or entirely explained by the increase in cytosolic Ca^2+^ concentration. As a bonus, α-lipoic acid is known to cause eryptosis by decreasing ATP levels and increasing ceramide synthesis. In addition, α-lipoic acid stimulates eryptosis by triggering caspases and generating oxidative stress. Because of its antioxidant qualities, α-lipoic acid is used for the treatment and prevention of several diseases. [51].
(e) Xenobiotics: Eryptosis can be triggered by several different types of xenobiotics. One such cause of eryptosis is cadmium poisoning, which increases the Ca^2+^ ion concentration in erythrocytes while decreasing the K^+^ ion concentration. This clarifies why some people who have been exposed to cadmium develop anemia [52]. In addition, exposure of erythrocytes to aluminum ions [53], hexavalent chromium [4], lithium [54], and the drug Azathioprine [55] have been reported to induce suicidal erythrocyte death by decreasing cytosolic ATP, increasing the intracellular Ca^2+^ ion concentration, activating cell membrane scrambling, and cell shrinkage.
(f) IgG Anti-A: It is also known that anti-A IgG antibodies enhance Ca^2+^ ion influx into erythrocytes, resulting in erythrocyte clearance. This is consistent with the immune system’s response to antigen A in autoimmune disorders and following an ABO blood transfusion [56].

Numerous compounds can prevent eryptosis, some of which are mentioned below.

(a) Erythropoietin: Erythropoietin promotes erythrocyte differentiation and protects erythrocytes by blocking eryptosis processes [38]. In direct opposition to the eryptosis process, erythropoietin blocks Ca^2+^-permeable cation channels [57].
(b) Xenobiotics: Many xenobiotics have been shown to suppress eryptosis. The antimicrobial agent thymol is a naturally occurring substance found in plants that prevents eryptosis by reducing cytosolic Ca^2+^ activity and preventing oxidative damage. However, it does not prevent the occurrence of cell shrinkage [58]. Flufenamic acid, a nonsteroidal anti-inflammatory medication, has been demonstrated to suppress eryptosis via altering Ca^2+^-permeable cation channels [59].
(c) Catecholamines: Certain catecholamines, including dopamine, epinephrine, and isoproterenol, are considered to inhibit eryptosis by impairing the Ca^2+^ cation channels’ ability to enhance the entry of Ca^2+^ ions [36]. The literature indicates that the amounts of catecholamines required to exert an anti-eryptotic impact are lower in the body than those required to produce these effects [60] Contrary to this, research has demonstrated that dopamine can be used to treat some disorders associated with erythrocyte toxicity by preventing the erythrocytes from entering eryptosis [36].
(d) Adenosine: Adenosine inhibits eryptosis by preventing Ca^2+^ entry and, therefore, Ca^2+^-dependent membrane scrambling and cell shrinkage [53]. It is also known that caffeine affects eryptosis via its effects on adenosine receptors, phosphodiesterases, channels, and intracellular Ca^2+^ release [61].
(e) Nitric oxide: Nitric oxide is produced in erythrocytes from deoxygenated hemoglobin and is known to suppress eryptosis because it promotes vasodilation in hypoxic regions by activating protein G kinase, which is required for erythrocyte sustenance and survival [48,62].

Inhibition of eryptosis is considered critical in some therapeutic situations since these compounds may be effective in treating individuals suffering from disorders characterized by eryptotic processes. Patients with sickle cell anemia or malaria experience an increase in eryptosis, which may result in further anemia. In such instances, it may be advantageous to provide eryptosis inhibitors in order to restore the erythrocyte equilibrium in the blood stream [2].

## 6. Pathophysiological Significance of Eryptosis

There are two important pathogenic effects of phosphatidylserine presentation on eryptotic cell surfaces: both phagocytosis and adhesion of erythrocytes to vascular endothelium cells, which express phosphatidylserine receptors, are mediated by phosphatidylserine [63,64].

Acute loss of erythrocytes, or anemia, may happen from excessive eryptosis that triggers the phagocytosis of numerous RBCs [16]. As such, many of the stimulators of eryptosis are linked to anemia, such that pharmacological medications that induce eryptosis are known to produce anemia as a side effect and eryptosis-associated disorders are mirrored or even defined by anemia [43].

It is possible that the microcirculation is impaired by eryptotic erythrocytes adhering to vascular endothelium cells, an effect potentially mediated via the phosphatidylserine receptor. As a result, eryptosis-stimulating agents may lead to microcirculation obstruction and a host of other cardiovascular side effects in addition to anemia [65]. It is possible, nevertheless, that inducing eryptosis will also have some positive outcomes. Plasmodium falciparum is a protozoan, a unicellular eukaryote, that causes malaria, a tropical illness that threatens hundreds of millions of people worldwide and is responsible for several hundred thousand fatalities annually. Erythrocytes are infected by a pathogen, which grows and develops inside the red cell before killing it and releasing new parasites. Malaria’s characteristic fever swings are brought on by the lysis [41,66]. Since parasite survival depends on erythrocytic maturation, preventing the parasite from reaching this stage by inducing eryptosis early may prove to be an effective treatment for this disease [41,48]. Resistance is a major problem with current malaria treatment methods that aim to kill the parasite that causes the disease. As a result, the treatment strategy of eryptosis activation may be useful in preventing resistance [41,48].

## 7. Role of Eryptosis in Disease

### 7.1. Nervous System

#### 7.1.1. Parkinson’s Disease

Eryptosis is known to be caused by a variety of factors, including oxidative stress, hyperosmotic shock, high temperatures, and energy deprivation [36,67]. The signaling molecules function as triggers, elevating cytoplasmic Ca^2+^ through Ca^2+^-permeable channels that have been activated [68].

It has been found that the production of both calpain and ceramide is changed in Parkinson’s disease (PD), and both of these signaling molecules play a crucial role in the development of eryptosis in the condition [69,70]. Two recent investigations have found that calpains are involved in the development of PD. The calpain family of calcium-dependent cysteine proteases are ubiquitous in mammalian tissues and play key roles in a variety of cellular processes, including axonal degeneration and apoptosis [71,72].

PD is linked to neurotoxins and Ca^2+^ homeostasis [73]. Synaptic dysfunction, decreased plasticity, and neuronal degeneration are all brought on by damage to cellular Ca^2+^-regulating systems in the plasma membrane, endoplasmic reticulum, and mitochondria [74], which is a hallmark of neurodegenerative diseases.

There is a dynamic ER-Ca^2+^-mitochondria linkage that may contribute to neuronal cell death in PD pathophysiology because of the intimate connection between the endoplasmic reticulum (ER) and mitochondria [75].

Ca^2+^ permeability is inhibited by nitric oxide (NO) and erythropoietin, which are two factors that may prevent eryptosis [50]. Nitrosylation of enzymes by NO and inhibition of Ca^2+^-induced phospholipid membrane scrambling occurs [8]. Accelerated eryptosis is balanced off by erythropoiesis and reticulocytosis [76].

Several abnormal signaling molecules have been linked to PD, and it is possible that they change the coagulation/hematology system, including RBC shape, in PD patients [70].

#### 7.1.2. Alzheimer’s Disease

Alzheimer’s disease (AD) is characterized by the presence of senile plaques in regions of the CNS where the neurodegenerative process occurs in afflicted patients. Amyloid β-peptide (Aβ), the main component of AD plaques, is neurotoxic, especially in aggregate form, and can cause death of neuronal cells. Aβ has been linked to oxidative stress. It may affect RBC metabolism and perhaps compromise RBC functioning and integrity, which might exacerbate vascular abnormalities that may contribute to AD. Eryptosis can be triggered by Aβ, which causes oxidative damage to red blood cells [77].

The toxic amyloid fragment Aß disrupts erythrocyte membrane phospholipids and reduces erythrocyte cell volume, at least in part as a result of ceramide formation. Aß may not only induce ceramide formation, but also stimulate cellular mechanisms that counteract glucose-induced eryptosis signaling, which involves protein kinase C activation. Enhanced eryptosis ought to expedite the clearance of circulating erythrocytes. Phosphatidylserine at the cell surface facilitates the binding of macrophage-expressed phosphatidylserine receptors. Binding to these receptors promotes the uptake and subsequent degradation of the exposed phosphatidylserine in erythrocytes. Erythrocytes exposed by the elimination of phosphatidylserine should result in anemia [37,38].

Anemia is a consequence of amyloidosis, which may theoretically occur from amyloid-induced eryptosis and subsequent clearance of eryptotic erythrocytes from the bloodstream. Phosphatidylserine-exposed erythrocytes may subsequently connect to receptors in the arterial wall, impeding the microcirculation and resulting in vascular problems, which are known to occur in Alzheimer’s disease [38].

### 7.2. Cardiovascular System

Cardiac insufficiency is frequently accompanied by anemia, which might have a deleterious influence on treatment outcomes [78]. Heart failure anemia may be caused by inflammation, renal dysfunction, or iron shortage [79]. Recent data suggests that anemia associated with cardiac insufficiency is caused by an increase in eryptosis [80]. When exposed to PS, patients with heart failure had lower cell volumes and higher erythrocyte concentrations. These patients’ erythrocytes generated more ROS, which may be a factor in their increased eryptosis. Heart failure pathogenesis is marked by elevated oxidative stress, which may lead to erythrocyte damage and eryptosis [80].

The risk of thrombosis is higher in those who are very obese [81]. Obese people are thought to experience rheological abnormalities and hypercoagulability as a result of increased erythrocyte aggregability and reduced erythrocyte deformability. Previous research indicated that erythrocyte PS exposure was considerably greater in individuals with a higher body mass index compared to healthy people, suggesting that eryptosis may contribute to the hypercoagulability associated with obesity [82,83]. Earlier research has shown that eryptosis raises the risk of thrombosis and cardiovascular disease, particularly in those who are overweight or obese [82,83]. Obesity has been shown to lower CD47 expression, an erythrocyte senescence marker. Erythrocyte dysfunction and membrane PS externalization were both significantly increased in mice fed a high-fat diet for an extended period. Atherosclerosis and macrophage activation were also observed in the obese animals, highlighting the relevance of a pathological nexus between erythrocytes, endothelial failure, and macrophage activation in obesity [84].

Arterial hypertension often coexists with dyslipidemia, both of which can cause oxidative stress, which induces eryptosis. Patients with hypertension, whether with or without dyslipidemia, had a higher rate of PS externalization associated with an increase in Ca^2+^ than normotensive patients. However, in normotensive patients with dyslipidemia, PS externalization was reported to be three times higher along with an increase in intracellular Ca^2+^ than in non-dyslipidemic patients, and the presence of hypertension doubles the difference [85]. This suggests that eryptosis might play a significant role in the pathophysiology of hypertension as well as dyslipidemia. To elucidate this possibility, it would be necessary to determine the cholesterol concentration and scramblase activity in erythrocytes from hypertensive dyslipidemic patients. Oxysterols, which are present in significant amounts in individuals with familial mixed hyperlipidemia, have been linked to PS externalization by raising the concentration of PGE2 in human erythrocytes in vitro, as shown by previous authors [86,87].

However, some studies have shown an unsuspected function of cholesterol in regulating phospholipid scrambling. According to these findings, cholesterol exchange between circulating plasma and erythrocyte membrane determines erythrocyte PS exposure. They reported that in cholesterol-depleted erythrocytes, PS exposure increases, while a cholesterol excess inhibits PS exposure, suggesting that cholesterol acts as a powerful scramblase inhibitor [88,89].

### 7.3. Immune System

Autoimmune hemolytic anemias (AIHA) are well-known disorders that can strike at any age when autoantibodies (aab) against red blood cells form (RBCs). Antibody classes IgG, IgM, and, less typically, IgA are represented by these aab. They react with RBCs at body temperature (warm aab) or <37 °C (cold aab). If complement is active, IgG aab can cause RBC destruction by Fc-mediated phagocytosis and C3b-mediated phagocytosis. C3b-mediated phagocytosis or the C5b-9 membrane assault complex are two mechanisms via which IgM aab might cause RBC destruction [90,91,92].

RBCs have been proven to commit to eryptosis in the same way as nucleated cells do throughout the previous two decades [93]. The breakdown of PS asymmetry by a variety of molecules after stimulation is thought to be the main cause of this process [5,93,94]. For the first time, RBCs from patients with severe cAIHA and, to a lesser extent, wAIHA, disclose PS as an eryptosis signal, according to one study. Their findings thus far suggest that only IgM and/or IgA aab may trigger eryptosis. This is confirmed by the fact that, despite significant hemolysis, none of the patients with AIHA owing to IgG heated aab showed eryptosis. Patients with AIHA who have eryptosis may benefit from therapy with eryptosis inhibitors, such as erythropoietin (EPO). Patients with cAIHA and wAIHA can now be effectively treated with EPO-stimulating drugs, according to new research [95,96].

#### COVID-19

Evidence linking SARS-CoV-2 and red blood cell physiology shows that the virus increases surface IgG levels, causing oxidative stress in RBCs, and a rise in intracellular Ca^2+^, all of which make RBCs more susceptible to damage from mechanical stress [97]. RBC deformability is known to diminish in the context of sepsis due to increased intracellular reactive oxygen species level, alterations that are analogous to those observed during eryptosis [65].

In the patient group, PS exposure has been found to be associated with an increase in D-dimers, suggesting the RBCs may play a role in the thrombotic mechanisms associated with COVID-19. Elevated intracellular Ca^2+^ also pushes membrane PS outwards, which promotes thrombosis by facilitating the formation and assembly of the prothrombinase complex and the production of microparticles [97].

Intact human erythrocytes may engulf SARS-CoV-2 and its active particles, transporting them to splenic and hepatic macrophages for clearance. The clearance of infectious agents attached to erythrocytes is a frequent defense mechanism. The attachment of erythrocytes to bacteria and viruses is facilitated by complement receptor 1, which also triggers phagocytosis and the clearance of the bacteria and viruses that have attached to the erythrocytes [98].

Several drugs, including glucocorticoids and anti-malaria medicines, such as chloroquine, are used to treat COVID-19. By boosting viral replication, chloroquine causes eryptosis and exacerbates virus-associated anemia [98].

### 7.4. Kidney Disease

Patients in the later stages of chronic renal impairment (stages G4 and G5) have greater eryptosis levels than those in the earlier stages of the illness (stage G1, G2 and G3) [99]. Erythrocytosis in chronic renal illness can be affected by a number of factors [100], including oxidative stress, energy loss, and uremic toxins. All these factors contribute to an increased rate of RBC mortality, and they all rise alongside a decline in renal function. Increased eryptosis has been linked to uremic toxins in patients with chronic renal disease [99]. Phosphatidylserine (PS) is exposed on the surface of RBCs because indoxyl sulfate raises the cytosolic calcium concentration and stimulates erythrocyte cell membrane scrambling [101]. Ceramide levels were shown to be elevated by indoxyl sulfate, which was previously established to have a role in eryptosis [101]. Increases in the cytosolic calcium concentration and eryptosis levels are also attributable to acrolein, which appears to induce ceramide production [102].

In addition, vanadate has been shown to cause eryptosis by blocking ATP synthesis, thus producing an energy-deficient condition in individuals with chronic renal disease [99]. Recent research has shown that uremic toxins, including urea and p-cresol, are cytotoxic to healthy RBCs. A lower glomerular filtration rate and a longer time spent on dialysis are both associated with higher levels of oxidative stress. As a result of aging, diabetes, hypertension, and dyslipidemia, as well as the suppression of antioxidant systems (lower levels of vitamin C and glutatione), oxidative stress is increased in end-stage renal disease [99].

Hyperglycemia specifically causes an increase in oxidative stress and ROS generation, both of which have a role in the development of diabetic nephropathy. Studies have demonstrated that oxidative stress and eryptosis are both exacerbated when diabetes and CKD coexist [103].

Renal ischemia and glomerular damage can be directly caused by oxidant species, which furthers renal damage. The process of hemodialysis itself is inflammatory and pro-inflammatory, leading to an increase in oxidative stress. It has also been recently shown that an individual’s risk of RBC mortality is increased when hypoxia and uremia coexist, which in turn contributes to the development of anemia in dialysis patients.

Damage to red blood cell membranes is a result of oxidative stress. Reactive oxygen species (ROS) cause damage to the lipids and proteins of RBC membranes, which in turn causes reorganization of the erythrocyte skeleton and a decrease in the membrane’s stability and deformability [104]. Finally, oxidative stress promotes microvesicle activity and caspase activation, both of which exacerbate eryptosis and renal anemia [100].

### 7.5. Digestive System

A variety of related diseases, including variceal hemorrhage, malignancy, viral infections, and chronic inflammation, and the depletion of important nutrients, such as vitamin B12 and folate, can cause anemia in patients with hepatic failure and fibrosis [105,106]. When the liver fails, anemia can result from rapid erythrocyte suicide death, which has been linked to higher bilirubin levels [107]. Blood levels of conjugated bilirubin promote ceramide formation and cytosolic Ca^2+^ activity, which both lead to increased PS exposure and erythrocyte clearance [107]. Cholestatic liver illness has been linked to hemolysis, increased intracellular Ca^2+^, ceramide production, and PS externalization in erythrocytes, all of which are triggered by elevated plasma levels of bile acids such glycochenodeoxycholate and taurochenodeoxycholate. Therefore, many variables may lead to anemia and a decreased erythrocyte lifespan in liver illness [107].

Wilson’s disease, a hereditary illness that causes Cu^2+^ buildup in cells and eventually leads to liver cirrhosis, has eryptosis as a significant contributor to the pathogenesis of anemia. Increased plasma levels of acid SM, which triggers ceramide production in both erythrocytes and hepatocytes, stimulate eryptosis in this scenario. Acid SM deficiency or pharmacological suppression has been demonstrated to reduce eryptosis and extend the longevity of rats susceptible to Wilson’s disease, a hereditary disorder. Cu^2+^-related oxidants in Wilson’s disease may lead to lower erythrocyte survival, at least in part [108].

### 7.6. Diabetes Mellitus

Diabetic individuals have a higher percentage of PS-exposed erythrocytes in their bloodstream [76]. Methylglyoxal, a byproduct of glycolysis, has been implicated with eryptotic syndrome. To some extent, methylglyoxal increases erythrocyte PS exposure by inhibiting glucose consumption, ATP synthesis, glutathione (GSH) creation, and anti-oxidative defense. Methylglyoxal’s action stimulates erythrocyte PS exposure, leading to anemia and/or diabetic microangiopathy in diabetic individuals [38]. Diabetes is associated with a weakened antioxidative defense, which can lead to cardiovascular problems [109]. Phospholipid scrambling of erythrocyte membranes is favored by diabetes patients’ enhanced superoxide dismutase activity and ROS generation. It has also been shown that high extracellular glucose concentrations in vitro can lead to an increase in Ca^2+^ and cation channel activity, which suggests that hyperglycemia has an adverse effect on cell viability. Erythrocyte dysfunction in diabetes can also be caused by processes independent of Ca^2+^, such as activation of caspase 3 [110].

### 7.7. Malignancy

It is typical for anemia to be present at various stages of cancer because of blood loss, reduced erythropoietin production or diminished erythropoietin efficiency, and depletion of critical nutrients [111,112]. Anemia was shown to be associated with elevated erythrocyte cytosolic Ca^2+^ activity, ceramide synthesis, ROS production, and PS exposure in a recent study of lung cancer patients [113]. In addition, the unfavorable effects of cytostatic therapy may confuse the pathophysiology of tumor-associated anemia [76]. Cancer patients may have anemia as a result of the use of chemotherapeutic medicines, such as paclitaxel, sorafenib, and sunitinib, as well as carmustine, estramustine, cisplatin, and mitotane in vitro and in vivo investigations [76,114,115,116].

Adenomatous polyposis coli (APC) gene loss-of-function mutations cause numerous colonic adenomas, which eventually progress to colon cancer. Intestinal cancers and severe anemia are seen in mice with faulty APCs [117]. However, erythrocytes from mice with anemia have been found to be more vulnerable to anemia-causing eryptosis than previously thought [36]. Splenomegaly is further exacerbated by an increase in the clearance of erythrocytes in the mice [36]. Even though increased cytosolic Ca^2+^ activity does not appear to be a factor in the development of accelerated eryptosis, erythrocytes from APC-deficient animals had lower ATP levels, suggesting that erythrocyte death is susceptible to an energy imbalance [36].

Myelodysplastic syndrome MDS, a clonal condition defined by persistently low levels of hemopoiesis (the production of red blood cells), causes erythrocytes to undergo elevated levels of oxidative stress and PS exposure, reducing their longevity in the bloodstream [118]. Patients with MDS are more likely to have PS exposure on younger and lighter erythrocytes than healthy persons [119]. Glyphophorin expression was shown to be elevated on the cell surface of young erythrocytes from MDS patients, which may have the effect of masking the increased PS exposure and preventing premature phagocytosis [119]. Chronic anemia in patients with myelodysplastic syndrome may have a role for stimulated eryptosis. In particular, an elevated incidence of thrombosis frequently complicates the natural course of MDS. This suggests that enhanced eryptosis in MDS may contribute to the increased prothrombotic risk associated with this disease [119].

### 7.8. Chronic Inflammatory Disease

One of the most prevalent causes of anemia in the elderly and chronically unwell is inflammation. Iron homeostasis and erythropoiesis inhibition by pro-inflammatory cytokines are the primary causes of anemia in inflammatory disorders [120]. Erythrocyte membrane changes resembling eryptosis were recently demonstrated to be induced by pro-inflammatory cytokines [121]. Arterial occlusion occurs as a result of arthritis, an inflammatory illness of the vascular wall, which can also produce variable degrees of anemia. Arteritis patients are more susceptible to anemia because of increased eryptosis, according to a recent study. Oxidative stress and cytosolic Ca^2+^ levels rise in tandem with increasing eryptosis. Ischemic vascular occlusion in individuals with arteritis may be facilitated by eryptotic blood cells adhering more strongly to the endothelial cells that line the blood vessels [122].

More than half of systemic lupus erythematosus (SLE) patients are anemic. Autoimmune erythrocyte destruction and immune-mediated hematopoietic failure are two plausible causes of anemia in SLE patients. SLE patients may also have antibodies against erythropoietin, a protein that promotes the formation of healthy erythrocytes [123]. Anemia in SLE patients can be attributed, at least in part, to increased Ca^2+^ influx and increased ROS abundance in erythrocytes, which contributes to eryptosis and reduced erythrocytes in SLE patients [124].

### 7.9. Aging

The incidence of anemia in the elderly rises with age and affects 50% of the population over the age of 80 years [125]. It is possible that anemia’s etiology is obscured in many older patients due to coexisting diseases. The PS-exposed erythrocyte percentage is higher in the elderly, which correlates with higher levels of oxidative stress, according to a recent study. However, increased cytoplasmic Ca^2+^ activity or ceramide signaling are not associated with increased eryptosis in the aged [126]. Increased eryptosis has been seen in mice lacking Klotho, an anti-aging membrane protein mostly found in the parathyroid glands, kidneys, and choroid plexus; this finding is remarkable. An animal model of accelerated aging has been developed using Klotho-deficient mice. In addition to increased erythrocyte turnover, erythrocytes from Klotho-deficient animals demonstrate increased vulnerability to eryptosis induced by energy deficiency and oxidative stress. It was shown that a vitamin D deficit diet reduced the impact of Klotho insufficiency on erythrocytes. A progeroid mouse model has mtDNA mutations that cause erythrocytes to have abnormal iron loading and oxidative stress, which leads to early death of the cells. Erythropoiesis may, therefore, have a role in age-related anemia, as evidenced by human research and animal models [127].

## 8. Prevention of Eryptosis by Natural Compounds

Some natural or phytochemical agents are efficacious ex vivo and in vivo as inhibitors of eryptosis, which, as previously stated, is related to many disorders [113,128]. In addition, these chemicals might be coupled with anticancer treatments, as the prevention of eryptosis could minimize the anemic condition brought on by chemotherapy [16]. By acting on distinct biomolecular targets, the structural variety of these compounds made it feasible to suppress the eryptosis generated by varied stimuli. Using several RBC ex vivo models and certain animal models, several studies assessed the primary pro-eryptotic signals, including PS externalization, rise in intracellular ROS and Ca^2+^, suppression of cellular reserves of GSH, change in cell volume, and caspase activation.

It has been demonstrated that natural compounds, including phenols, alkaloids, and others, can cure and prevent oxidative stress and inflammation [129,130]. Consequently, a crucial step in the therapy and/or co-adjuvant strategies for eryptosis-related disorders might be to delve more into their function in the battle against eryptosis.

**Figure 4:**
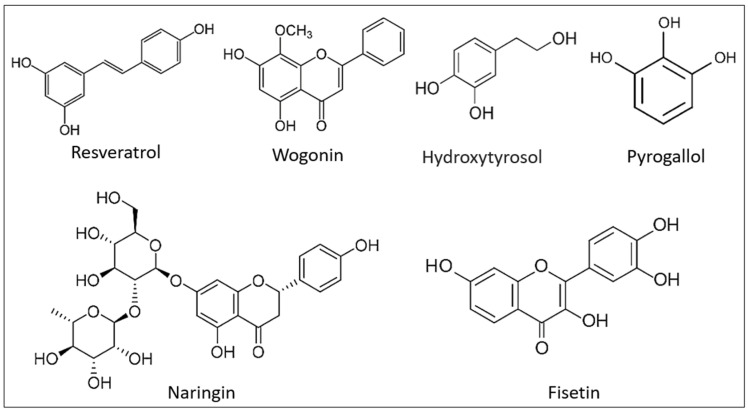
Chemical structure of some polyphenolic compounds with anti-eryptotic activity.

Phenolic compounds (PCs) are a class of phytochemicals present in many different plant tissues. They are especially popular among people who follow the Mediterranean diet, which emphasizes the consumption of cereals, various fruit and vegetable species, and olive oil [131]. PCs possess several bioactive qualities and, despite the fact that they are not nutritive, their consumption provides beneficial health effects, such as antioxidant effects, that assist to prevent the progression of several major illnesses, including cancer, Alzheimer’s, and diabetes [132]. Many studies show that PCs are advantageous, especially for Alzheimer’s disease, through their ability to interact with transition metals, neutralize free radicals, suppress inflammation, control the activity of enzymes, alter intracellular signaling networks, and modulate gene expression [133]. Since 2009, several phenols, including resveratrol, cinnamonaldehyde, hydroxytyrosol, pyrrogallol, Naringin, Fisetin, and Wogonin, have been studied for their anti- eryptotic activity (Figure 4). Based on the data evaluated, several phenols share numerous characteristics that inhibit eryptosis triggered by specific stimuli [134].

**Figure 5:**
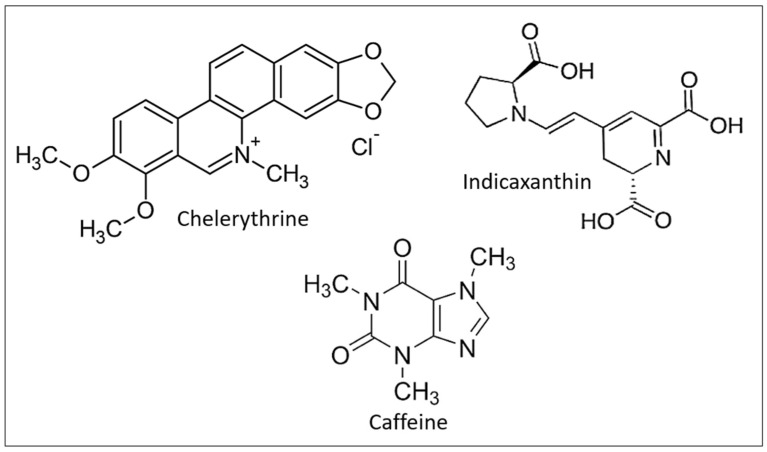
Chemical structure of some alkaloid compounds with anti-eryptotic activity.

Alkaloid compounds (ACs) are typically found in plants, fungi, and bacteria as a type of nitrogen-containing chemical molecules. They exhibit considerable biological properties and are frequently one of the most essential active components in phytotherapy. Dicotyledons, which are higher plants, contain the bulk of alkaloids. The development of alkaloid chemistry has progressed as a result of developments in the separation of natural products and the creation of new technologies and techniques [135]. Alkaloids may be categorized based on their origins and chemical structures, and they are mostly utilized as analgesics, cough suppressants, muscle relaxants, antimicrobials, and precursors of semisynthetic medicines [136]. In 2008, the first investigation on the anti-eryptotic action of ACs was published. Lang et al. demonstrated that caffeine, at a concentration range of 50–500 µM, protects against eryptosis produced by energy depletion and cell shrinkage by inhibiting the externalization of PS and restoring normal levels of Forward scatter (FSC) and intracellular Ca^2+^ [61]. A number of other ACs, by countering oxidative stress and intracellular Ca^2+^, have been reported to have anti-eryptotic properties (Figure 5) [134].

The natural world is full of a wide variety of additional substances that have the ability to inhibit the eryptosis caused by a variety of different triggers. One of them is vitamin C (VitC), which, by inserting into the membrane and neutralizing oxidative stress, lowers energy depletion, oxidative stress, and cell shrinkage [137]. A variety of other natural compounds, including, L-carnitine, plant sterols, and cinnamaldehyde, by countering oxidative stress and restoring normal calcium levels in the cells, inhibit eryptosis [134].

## 9. Conclusions

Eryptosis is an important process characterized by loss of ionic regulation, cell shrinkage, membrane blebbing, and disruption of phospholipid organization in the cell membrane. The physiological relevance of eryptosis is that it provides an avenue to eliminate injured, aged, or infected erythrocytes from the systemic circulation and thus safeguards against intravascular hemolysis. Chronic inflammation, sepsis, malignancy, uremia, and hepatic failure are only few of the human disorders associated with excessive eryptosis. A network of ion channels, membrane proteins, and intracellular enzymes regulate eryptosis, which is triggered by several pathophysiological mechanisms as well as a wide assortment of endogenous compounds and xenobiotics. Similarly, a growing number of compounds, such as erythropoietin, nitric oxide, thymol, and catecholamines, have been identified as inhibitors of eryptosis with promising therapeutic implications. There is a well-defined but poorly understood molecular cross-talk between erythrocytes and phagocytic cells through which eryptotic cells are identified and engulfed by macrophages and dendritic cells prior to their destruction in the reticulo-endothelial system. Efforts must be directed toward the identification and characterization of novel modulators of eryptosis especially within the context of therapeutic development. Equally important is the need for a clearer understanding of the clinical utility of targeting erythrocyte survival in pathological conditions.

## Data Availability

Not applicable.

## References

[1] Vahedi A., Bigdelou P., Farnoud A.M. (2020). Quantitative analysis of red blood cell membrane phospholipids and modulation of cell-macrophage interactions using cyclodextrins. Sci. Rep..

[2] Repsold L., Joubert A.M. (2018). Eryptosis: An Erythrocyte’s Suicidal Type of Cell Death. Biomed. Res. Int..

[3] Sultan S.A., Khawaji M.H., Alsughayyir J., Alfhili M.A., Alamri H.S., Alrfaei B.M. (2020). Antileukemic activity of sulfoxide nutraceutical allicin against THP-1 cells is associated with premature phosphatidylserine exposure in human erythrocytes. Saudi J. Biol. Sci..

[4] Lupescu A., Shaik N., Jilani K., Zelenak C., Lang E., Pasham V., Zbidah M., Plate A., Bitzer M., Foller M. (2012). Enhanced erythrocyte membrane exposure of phosphatidylserine following sorafenib treatment: An in vivo and in vitro study. Cell Physiol. Biochem..

[5] Pretorius E., du Plooy J.N., Bester J. (2016). A Comprehensive Review on Eryptosis. Cell Physiol. Biochem..

[6] Alfhili M.A., Alamri H.S., Alsughayyir J., Basudan A.M. (2022). Induction of hemolysis and eryptosis by occupational pollutant nickel chloride is mediated through calcium influx and p38 MAP kinase signaling. Int. J. Occup. Med. Environ. Health.

[7] Gatidis S., Zelenak C., Fajol A., Lang E., Jilani K., Michael D., Qadri S.M., Lang F. (2011). p38 MAPK activation and function following osmotic shock of erythrocytes. Cell Physiol. Biochem..

[8] Lang F., Lang E., Foller M. (2012). Physiology and pathophysiology of eryptosis. Transfus. Med. Hemother..

[9] Alfhili M.A., Lee M.H. (2021). Flow Cytofluorometric Analysis of Molecular Mechanisms of Premature Red Blood Cell Death. Methods Mol. Biol..

[10] Al Mamun Bhuyan A., Lang F. (2018). Stimulation of Eryptosis by Afatinib. Cell Physiol. Biochem..

[11] Gbotosho G.O., Okuboyejo T., Happi C.T., Sowunmi A. (2014). Fall in hematocrit per 1000 parasites cleared from peripheral blood: A simple method for estimating drug-related fall in hematocrit after treatment of malaria infections. Am. J. Ther..

[12] Alfhili M.A., Basudan A.M., Alsughayyir J. (2021). Antiproliferative Wnt inhibitor wogonin prevents eryptosis following ionophoric challenge, hyperosmotic shock, oxidative stress, and metabolic deprivation. J. Food Biochem..

[13] Foller M., Lang F. (2020). Ion Transport in Eryptosis, the Suicidal Death of Erythrocytes. Front. Cell Dev. Biol..

[14] Sathi A., Viswanad V., Aneesh T.P., Kumar B.A. (2014). Pros and cons of phospholipid asymmetry in erythrocytes. J. Pharm. Bioallied Sci..

[15] Bissinger R., Bhuyan A.A.M., Qadri S.M., Lang F. (2019). Oxidative stress, eryptosis and anemia: A pivotal mechanistic nexus in systemic diseases. FEBS J..

[16] Lang F., Bissinger R., Abed M., Artunc F. (2017). Eryptosis-the Neglected Cause of Anemia in End Stage Renal Disease. Kidney Blood Press Res..

[17] Barodka V.M., Nagababu E., Mohanty J.G., Nyhan D., Berkowitz D.E., Rifkind J.M., Strouse J.J. (2014). New insights provided by a comparison of impaired deformability with erythrocyte oxidative stress for sickle cell disease. Blood Cells Mol. Dis..

[18] Dreischer P., Duszenko M., Stein J., Wieder T. (2022). Eryptosis: Programmed Death of Nucleus-Free, Iron-Filled Blood Cells. Cells.

[19] Akiel M., Alsughayyir J., Basudan A.M., Alamri H.S., Dera A., Barhoumi T., Al Subayyil A.M., Basmaeil Y.S., Aldakheel F.M., Alakeel R. (2021). Physcion Induces Hemolysis and Premature Phosphatidylserine Externalization in Human Erythrocytes. Biol. Pharm. Bull..

[20] Restivo I., Attanzio A., Tesoriere L., Allegra M. (2021). Suicidal Erythrocyte Death in Metabolic Syndrome. Antioxidants.

[21] Pretini V., Koenen M.H., Kaestner L., Fens M., Schiffelers R.M., Bartels M., Van Wijk R. (2019). Red Blood Cells: Chasing Interactions. Front. Physiol..

[22] Vahedi-Mazdabadi Y., Karimpour-Razkenari E., Akbarzadeh T., Lotfian H., Toushih M., Roshanravan N., Saeedi M., Ostadrahimi A. (2020). Anti-cholinesterase and Neuroprotective Activities of Sweet and Bitter Apricot Kernels (*Prunus armeniaca* L.). Iran J. Pharm. Res..

[23] Ran Q., Xiang Y., Liu Y., Xiang L., Li F., Deng X., Xiao Y., Chen L., Chen L., Li Z. (2015). Eryptosis Indices as a Novel Predictive Parameter for Biocompatibility of Fe3O4 Magnetic Nanoparticles on Erythrocytes. Sci. Rep..

[24] Ohvo-Rekila H., Ramstedt B., Leppimaki P., Slotte J.P. (2002). Cholesterol interactions with phospholipids in membranes. Prog. Lipid. Res..

[25] Mohandas N., Gallagher P.G. (2008). Red cell membrane: Past, present, and future. Blood.

[26] Salzer U., Prohaska R. (2001). Stomatin, flotillin-1, and flotillin-2 are major integral proteins of erythrocyte lipid rafts. Blood.

[27] van Meer G., Voelker D.R., Feigenson G.W. (2008). Membrane lipids: Where they are and how they behave. Nat. Rev. Mol. Cell Biol..

[28] Sirachainan N., Thongsad J., Pakakasama S., Hongeng S., Chuansumrit A., Kadegasem P., Tirakanjana A., Archararit N., Sirireung S. (2012). Normalized coagulation markers and anticoagulation proteins in children with severe beta-thalassemia disease after stem cell transplantation. Thromb. Res..

[29] Weiss E., Cytlak U.M., Rees D.C., Osei A., Gibson J.S. (2012). Deoxygenation-induced and Ca(2+) dependent phosphatidylserine externalisation in red blood cells from normal individuals and sickle cell patients. Cell Calcium.

[30] Pomorski T., Menon A.K. (2006). Lipid flippases and their biological functions. Cell Mol. Life Sci..

[31] Lux S.E.t. (2016). Anatomy of the red cell membrane skeleton: Unanswered questions. Blood.

[32] Barbarino F., Waschenbach L., Cavalho-Lemos V., Dillenberger M., Becker K., Gohlke H., Cortese-Krott M.M. (2021). Targeting spectrin redox switches to regulate the mechanoproperties of red blood cells. Biol. Chem..

[33] Ghashghaeinia M., Cluitmans J.C., Akel A., Dreischer P., Toulany M., Koberle M., Skabytska Y., Saki M., Biedermann T., Duszenko M. (2012). The impact of erythrocyte age on eryptosis. Br. J. Haematol..

[34] Lang K.S., Lang P.A., Bauer C., Duranton C., Wieder T., Huber S.M., Lang F. (2005). Mechanisms of suicidal erythrocyte death. Cell Physiol. Biochem..

[35] Berg C.P., Engels I.H., Rothbart A., Lauber K., Renz A., Schlosser S.F., Schulze-Osthoff K., Wesselborg S. (2001). Human mature red blood cells express caspase-3 and caspase-8, but are devoid of mitochondrial regulators of apoptosis. Cell Death Differ..

[36] Qadri S.M., Mahmud H., Lang E., Gu S., Bobbala D., Zelenak C., Jilani K., Siegfried A., Foller M., Lang F. (2012). Enhanced suicidal erythrocyte death in mice carrying a loss-of-function mutation of the adenomatous polyposis coli gene. J. Cell Mol. Med..

[37] Boas F.E., Forman L., Beutler E. (1998). Phosphatidylserine exposure and red cell viability in red cell aging and in hemolytic anemia. Proc. Natl. Acad. Sci. USA.

[38] Nicolay J.P., Schneider J., Niemoeller O.M., Artunc F., Portero-Otin M., Haik G., Thornalley P.J., Schleicher E., Wieder T., Lang F. (2006). Stimulation of suicidal erythrocyte death by methylglyoxal. Cell Physiol. Biochem..

[39] Clarke R.J., Hossain K.R., Cao K. (2020). Physiological roles of transverse lipid asymmetry of animal membranes. Biochim. Biophys. Acta Biomembr..

[40] Kuhn V., Diederich L., Keller T.C.S.t., Kramer C.M., Luckstadt W., Panknin C., Suvorava T., Isakson B.E., Kelm M., Cortese-Krott M.M. (2017). Red Blood Cell Function and Dysfunction: Redox Regulation, Nitric Oxide Metabolism, Anemia. Antioxid. Redox Signal..

[41] Boulet C., Doerig C.D., Carvalho T.G. (2018). Manipulating Eryptosis of Human Red Blood Cells: A Novel Antimalarial Strategy?. Front. Cell Infect. Microbiol..

[42] Bryer E., Henry D. (2018). Isolated hypoglossal nerve palsy as a presenting symptom of metastatic peripheral T-cell lymphoma-not otherwise specified (PTCL-NOS): A unique case & a review of the literature. Int. J. Hematol. Oncol..

[43] Lang E., Lang F. (2015). Mechanisms and pathophysiological significance of eryptosis, the suicidal erythrocyte death. Semin. Cell Dev. Biol..

[44] Alfhili M.A., Aljuraiban G.S. (2021). Lauric Acid, a Dietary Saturated Medium-Chain Fatty Acid, Elicits Calcium-Dependent Eryptosis. Cells.

[45] Alsughayyir J., Alshaiddi W., Alsubki R., Alshammary A., Basudan A.M., Alfhili M.A. (2022). Geraniin inhibits whole blood IFN-gamma and IL-6 and promotes IL-1beta and IL-8, and stimulates calcium-dependent and sucrose-sensitive erythrocyte death. Toxicol. Appl. Pharmacol..

[46] Kim-Shapiro D.B., Gladwin M.T. (2014). Mechanisms of nitrite bioactivation. Nitric Oxide.

[47] Ren G., Roberts A.I., Shi Y. (2011). Adhesion molecules: Key players in Mesenchymal stem cell-mediated immunosuppression. Cell Adh. Migr..

[48] Foller M., Huber S.M., Lang F. (2008). Erythrocyte programmed cell death. IUBMB Life.

[49] Lang E., Bissinger R., Qadri S.M., Lang F. (2017). Suicidal death of erythrocytes in cancer and its chemotherapy: A potential target in the treatment of tumor-associated anemia. Int. J. Cancer.

[50] Foller M., Braun M., Qadri S.M., Lang E., Mahmud H., Lang F. (2010). Temperature sensitivity of suicidal erythrocyte death. Eur. J. Clin. Investig..

[51] Kucherenko Y.V., Bhavsar S.K., Grischenko V.I., Fischer U.R., Huber S.M., Lang F. (2010). Increased cation conductance in human erythrocytes artificially aged by glycation. J. Membr. Biol..

[52] Sopjani M., Foller M., Dreischer P., Lang F. (2008). Stimulation of eryptosis by cadmium ions. Cell Physiol. Biochem..

[53] Niemoeller O.M., Kiedaisch V., Dreischer P., Wieder T., Lang F. (2006). Stimulation of eryptosis by aluminium ions. Toxicol. Appl. Pharmacol.

[54] Nicolay J.P., Gatz S., Lang F., Lang U.E. (2010). Lithium-induced suicidal erythrocyte death. J. Psychopharmacol..

[55] Geiger C., Foller M., Herrlinger K.R., Lang F. (2008). Azathioprine-induced suicidal erythrocyte death. Inflamm. Bowel. Dis..

[56] Attanasio P., Shumilina E., Hermle T., Kiedaisch V., Lang P.A., Huber S.M., Wieder T., Lang F. (2007). Stimulation of eryptosis by anti-A IgG antibodies. Cell Physiol. Biochem..

[57] Kiefer C.R., Snyder L.M. (2000). Oxidation and erythrocyte senescence. Curr. Opin. Hematol..

[58] Mahmud H., Mauro D., Foller M., Lang F. (2009). Inhibitory effect of thymol on suicidal erythrocyte death. Cell Physiol. Biochem..

[59] Kasinathan R.S., Foller M., Koka S., Huber S.M., Lang F. (2007). Inhibition of eryptosis and intraerythrocytic growth of Plasmodium falciparum by flufenamic acid. Naunyn Schmiedebergs Arch. Pharmacol..

[60] Lang P.A., Kempe D.S., Akel A., Klarl B.A., Eisele K., Podolski M., Hermle T., Niemoeller O.M., Attanasio P., Huber S.M. (2005). Inhibition of erythrocyte “apoptosis” by catecholamines. Naunyn Schmiedebergs Arch. Pharmacol..

[61] Floride E., Foller M., Ritter M., Lang F. (2008). Caffeine inhibits suicidal erythrocyte death. Cell Physiol. Biochem..

[62] Lang F., Gulbins E., Lerche H., Huber S.M., Kempe D.S., Foller M. (2008). Eryptosis, a window to systemic disease. Cell Physiol. Biochem..

[63] Setty B.N., Betal S.G. (2008). Microvascular endothelial cells express a phosphatidylserine receptor: A functionally active receptor for phosphatidylserine-positive erythrocytes. Blood.

[64] Wautier M.P., Heron E., Picot J., Colin Y., Hermine O., Wautier J.L. (2011). Red blood cell phosphatidylserine exposure is responsible for increased erythrocyte adhesion to endothelium in central retinal vein occlusion. J. Thromb. Haemost..

[65] Pretorius E. (2018). Erythrocyte deformability and eryptosis during inflammation, and impaired blood rheology. Clin. Hemorheol. Microcirc..

[66] Foller M., Bobbala D., Koka S., Huber S.M., Gulbins E., Lang F. (2009). Suicide for survival--death of infected erythrocytes as a host mechanism to survive malaria. Cell Physiol. Biochem..

[67] Lang F., Abed M., Lang E., Foller M. (2014). Oxidative stress and suicidal erythrocyte death. Antioxid. Redox Signal..

[68] Gao M., Lau P.M., Kong S.K. (2014). Mitochondrial toxin betulinic acid induces in vitro eryptosis in human red blood cells through membrane permeabilization. Arch. Toxicol..

[69] Funke C., Schneider S.A., Berg D., Kell D.B. (2013). Genetics and iron in the systems biology of Parkinson’s disease and some related disorders. Neurochem. Int..

[70] Pretorius E., Swanepoel A.C., Buys A.V., Vermeulen N., Duim W., Kell D.B. (2014). Eryptosis as a marker of Parkinson’s disease. Aging.

[71] Samantaray S., Knaryan V.H., Shields D.C., Banik N.L. (2013). Critical role of calpain in spinal cord degeneration in Parkinson’s disease. J. Neurochem..

[72] Diepenbroek M., Casadei N., Esmer H., Saido T.C., Takano J., Kahle P.J., Nixon R.A., Rao M.V., Melki R., Pieri L. (2014). Overexpression of the calpain-specific inhibitor calpastatin reduces human alpha-Synuclein processing, aggregation and synaptic impairment in [A30P]alphaSyn transgenic mice. Hum. Mol. Genet..

[73] Arshad A., Chen X., Cong Z., Qing H., Deng Y. (2014). TRPC1 protects dopaminergic SH-SY5Y cells from MPP+, salsolinol, and N-methyl-(R)-salsolinol-induced cytotoxicity. Acta Biochim. Biophys. Sin.

[74] Mattson M.P. (2007). Calcium and neurodegeneration. Aging Cell.

[75] Arduino D.M., Esteves A.R., Cardoso S.M., Oliveira C.R. (2009). Endoplasmic reticulum and mitochondria interplay mediates apoptotic cell death: Relevance to Parkinson’s disease. Neurochem. Int..

[76] Lang F., Qadri S.M. (2012). Mechanisms and significance of eryptosis, the suicidal death of erythrocytes. Blood Purif..

[77] Carelli-Alinovi C., Misiti F. (2017). Erythrocytes as Potential Link between Diabetes and Alzheimer’s Disease. Front. Aging Neurosci..

[78] Uscinska E., Idzkowska E., Sobkowicz B., Musial W.J., Tycinska A.M. (2015). Anemia in Intensive Cardiac Care Unit patients—An underestimated problem. Adv. Med. Sci..

[79] von Haehling S., Jankowska E.A., Ponikowski P., Anker S.D. (2011). Anemia in heart failure: An overview of current concepts. Future Cardiol..

[80] Attanasio P., Bissinger R., Haverkamp W., Pieske B., Wutzler A., Lang F. (2015). Enhanced suicidal erythrocyte death in acute cardiac failure. Eur. J. Clin. Investig..

[81] Blokhin I.O., Lentz S.R. (2013). Mechanisms of thrombosis in obesity. Curr. Opin. Hematol..

[82] Sola E., Vaya A., Martinez M., Moscardo A., Corella D., Santaolaria M.L., Espana F., Hernandez-Mijares A. (2009). Erythrocyte membrane phosphatidylserine exposure in obesity. Obesity.

[83] Wiewiora M., Piecuch J., Sedek L., Mazur B., Sosada K. (2017). The effects of obesity on CD47 expression in erythrocytes. Cytom. B Clin. Cytom..

[84] Unruh D., Srinivasan R., Benson T., Haigh S., Coyle D., Batra N., Keil R., Sturm R., Blanco V., Palascak M. (2015). Red Blood Cell Dysfunction Induced by High-Fat Diet: Potential Implications for Obesity-Related Atherosclerosis. Circulation.

[85] Pinzon-Diaz C.E., Calderon-Salinas J.V., Rosas-Flores M.M., Hernandez G., Lopez-Betancourt A., Quintanar-Escorza M.A. (2018). Eryptosis and oxidative damage in hypertensive and dyslipidemic patients. Mol. Cell Biochem..

[86] Arca M., Natoli S., Micheletta F., Riggi S., Di Angelantonio E., Montali A., Antonini T.M., Antonini R., Diczfalusy U., Iuliano L. (2007). Increased plasma levels of oxysterols, in vivo markers of oxidative stress, in patients with familial combined hyperlipidemia: Reduction during atorvastatin and fenofibrate therapy. Free Radic. Biol. Med..

[87] Tesoriere L., Attanzio A., Allegra M., Cilla A., Gentile C., Livrea M.A. (2014). Oxysterol mixture in hypercholesterolemia-relevant proportion causes oxidative stress-dependent eryptosis. Cell Physiol. Biochem..

[88] Gottlieb M.H. (1980). Rates of cholesterol exchange between human erythrocytes and plasma lipoproteins. Biochim. Biophys. Acta.

[89] van Zwieten R., Bochem A.E., Hilarius P.M., van Bruggen R., Bergkamp F., Hovingh G.K., Verhoeven A.J. (2012). The cholesterol content of the erythrocyte membrane is an important determinant of phosphatidylserine exposure. Biochim. Biophys. Acta.

[90] Barcellini W. (2015). New Insights in the Pathogenesis of Autoimmune Hemolytic Anemia. Transfus. Med. Hemother..

[91] Packman C.H. (2015). The Clinical Pictures of Autoimmune Hemolytic Anemia. Transfus. Med. Hemother..

[92] Wouters D., Zeerleder S. (2015). Complement inhibitors to treat IgM-mediated autoimmune hemolysis. Haematologica.

[93] Bevers E.M., Williamson P.L. (2016). Getting to the Outer Leaflet: Physiology of Phosphatidylserine Exposure at the Plasma Membrane. Physiol. Rev..

[94] Nagata S., Suzuki J., Segawa K., Fujii T. (2016). Exposure of phosphatidylserine on the cell surface. Cell Death Differ..

[95] Salama A., Hartnack D., Lindemann H.W., Lange H.J., Rummel M., Loew A. (2014). The effect of erythropoiesis-stimulating agents in patients with therapy-refractory autoimmune hemolytic anemia. Transfus. Med. Hemother..

[96] Bartolmas T., Mayer B., Balola A.H., Salama A. (2018). Eryptosis in autoimmune haemolytic anaemia. Eur. J. Haematol..

[97] Bouchla A., Kriebardis A.G., Georgatzakou H.T., Fortis S.P., Thomopoulos T.P., Lekkakou L., Markakis K., Gkotzias D., Panagiotou A., Papageorgiou E.G. (2021). Red Blood Cell Abnormalities as the Mirror of SARS-CoV-2 Disease Severity: A Pilot Study. Front. Physiol..

[98] Ghashghaeinia M., Dreischer P., Wieder T., Koberle M. (2020). Coronavirus disease 2019 (COVID-19), human erythrocytes and the PKC-alpha/-beta inhibitor chelerythrine -possible therapeutic implication. Cell Cycle.

[99] Clementi A., Virzi G.M., Milan Manani S., Battaglia G.G., Ronco C., Zanella M. (2022). Eryptosis in Patients with Chronic Kidney Disease: A Possible Relationship with Oxidative Stress and Inflammatory Markers. J. Clin. Med..

[100] Li D., Zheng X., Zhang Y., Li X., Chen X., Yin Y., Hu J., Li J., Guo M., Wang X. (2022). What Should Be Responsible for Eryptosis in Chronic Kidney Disease?. Kidney Blood Press Res..

[101] Alzoubi K., Honisch S., Abed M., Lang F. (2013). Triggering of suicidal erythrocyte death by penta-O-galloyl-beta-D-glucose. Toxins.

[102] Ahmed M.S., Langer H., Abed M., Voelkl J., Lang F. (2013). The uremic toxin acrolein promotes suicidal erythrocyte death. Kidney Blood Press Res..

[103] Vodosek Hojs N., Bevc S., Ekart R., Hojs R. (2020). Oxidative Stress Markers in Chronic Kidney Disease with Emphasis on Diabetic Nephropathy. Antioxidants.

[104] Sudnitsyna J., Skverchinskaya E., Dobrylko I., Nikitina E., Gambaryan S., Mindukshev I. (2020). Microvesicle Formation Induced by Oxidative Stress in Human Erythrocytes. Antioxidants.

[105] Maheshwari A., Mishra R., Thuluvath P.J. (2004). Post-liver-transplant anemia: Etiology and management. Liver Transpl..

[106] Marks P.W. (2013). Hematologic manifestations of liver disease. Semin. Hematol..

[107] Lang E., Gatidis S., Freise N.F., Bock H., Kubitz R., Lauermann C., Orth H.M., Klindt C., Schuier M., Keitel V. (2015). Conjugated bilirubin triggers anemia by inducing erythrocyte death. Hepatology.

[108] Lang F., Huber S.M., Szabo I., Gulbins E. (2007). Plasma membrane ion channels in suicidal cell death. Arch. Biochem. Biophys..

[109] Soma P., Pretorius E. (2015). Interplay between ultrastructural findings and atherothrombotic complications in type 2 diabetes mellitus. Cardiovasc. Diabetol..

[110] Mohammedi K., Bellili-Munoz N., Marklund S.L., Driss F., Le Nagard H., Patente T.A., Fumeron F., Roussel R., Hadjadj S., Marre M. (2015). Plasma extracellular superoxide dismutase concentration, allelic variations in the SOD3 gene and risk of myocardial infarction and all-cause mortality in people with type 1 and type 2 diabetes. Cardiovasc. Diabetol..

[111] Gaspar B.L., Sharma P., Das R. (2015). Anemia in malignancies: Pathogenetic and diagnostic considerations. Hematology.

[112] Gilreath J.A., Stenehjem D.D., Rodgers G.M. (2014). Diagnosis and treatment of cancer-related anemia. Am. J. Hematol..

[113] Qadri S.M., Bissinger R., Solh Z., Oldenborg P.A. (2017). Eryptosis in health and disease: A paradigm shift towards understanding the (patho)physiological implications of programmed cell death of erythrocytes. Blood Rev..

[114] Arnold M., Bissinger R., Lang F. (2014). Mitoxantrone-induced suicidal erythrocyte death. Cell Physiol. Biochem..

[115] Jacobi J., Lang E., Bissinger R., Frauenfeld L., Modicano P., Faggio C., Abed M., Lang F. (2014). Stimulation of erythrocyte cell membrane scrambling by mitotane. Cell Physiol. Biochem..

[116] Peng Y., Zhang X., Feng X., Fan X., Jin Z. (2017). The crosstalk between microRNAs and the Wnt/beta-catenin signaling pathway in cancer. Oncotarget.

[117] Hodgson A., Wier E.M., Fu K., Sun X., Wan F. (2016). Ultrasound imaging of splenomegaly as a proxy to monitor colon tumor development in Apc(min716/+) mice. Cancer Med..

[118] Stein R.S. (2003). The role of erythropoietin in the anemia of myelodysplastic syndrome. Clin. Lymphoma.

[119] Basu S., Banerjee D., Ghosh M., Chakrabarti A. (2010). Erythrocyte membrane defects and asymmetry in paroxysmal nocturnal hemoglobinuria and myelodysplastic syndrome. Hematology.

[120] Abioye A.I., Park S., Ripp K., McDonald E.A., Kurtis J.D., Wu H., Pond-Tor S., Sharma S., Ernerudh J., Baltazar P. (2018). Anemia of Inflammation during Human Pregnancy Does Not Affect Newborn Iron Endowment. J. Nutr..

[121] Bester J., Pretorius E. (2016). Effects of IL-1beta, IL-6 and IL-8 on erythrocytes, platelets and clot viscoelasticity. Sci. Rep..

[122] Bissinger R., Kempe-Teufel D.S., Honisch S., Qadri S.M., Randrianarisoa E., Haring H.U., Henes J., Lang F. (2016). Stimulated Suicidal Erythrocyte Death in Arteritis. Cell. Physiol. Biochem..

[123] Giannouli S., Voulgarelis M., Ziakas P.D., Tzioufas A.G. (2006). Anaemia in systemic lupus erythematosus: From pathophysiology to clinical assessment. Ann. Rheum. Dis..

[124] Jiang P., Bian M., Ma W., Liu C., Yang P., Zhu B., Xu Y., Zheng M., Qiao J., Shuai Z. (2016). Eryptosis as an Underlying Mechanism in Systemic Lupus Erythematosus-Related Anemia. Cell Physiol. Biochem..

[125] Artz A.S., Thirman M.J. (2011). Unexplained anemia predominates despite an intensive evaluation in a racially diverse cohort of older adults from a referral anemia clinic. J. Gerontol. A Biol. Sci. Med. Sci..

[126] Lupescu A., Bissinger R., Goebel T., Salker M.S., Alzoubi K., Liu G., Chirigiu L., Mack A.F., Qadri S.M., Lang F. (2015). Enhanced suicidal erythrocyte death contributing to anemia in the elderly. Cell Physiol. Biochem..

[127] Kempe D.S., Ackermann T.F., Fischer S.S., Koka S., Boini K.M., Mahmud H., Foller M., Rosenblatt K.P., Kuro O.M., Lang F. (2009). Accelerated suicidal erythrocyte death in Klotho-deficient mice. Pflugers Arch..

[128] Alfhili M.A., Alsughayyir J., Basudan A.B. (2021). Epidemic dropsy toxin, sanguinarine chloride, stimulates sucrose-sensitive hemolysis and breakdown of membrane phospholipid asymmetry in human erythrocytes. Toxicon.

[129] Cilla A., Alegria A., Attanzio A., Garcia-Llatas G., Tesoriere L., Livrea M.A. (2017). Dietary phytochemicals in the protection against oxysterol-induced damage. Chem. Phys. Lipids.

[130] Yu M., Gouvinhas I., Rocha J., Barros A. (2021). Phytochemical and antioxidant analysis of medicinal and food plants towards bioactive food and pharmaceutical resources. Sci. Rep..

[131] Perez-Jimenez J., Neveu V., Vos F., Scalbert A. (2010). Identification of the 100 richest dietary sources of polyphenols: An application of the Phenol-Explorer database. Eur. J. Clin. Nutr..

[132] Albuquerque B.R., Heleno S.A., Oliveira M., Barros L., Ferreira I. (2021). Phenolic compounds: Current industrial applications, limitations and future challenges. Food Funct..

[133] Colizzi C. (2019). The protective effects of polyphenols on Alzheimer’s disease: A systematic review. Alzheimers Dement..

[134] Restivo I., Attanzio A., Tesoriere L., Allegra M., Garcia-Llatas G., Cilla A. (2022). Anti-Eryptotic Activity of Food-Derived Phytochemicals and Natural Compounds. Int. J. Mol. Sci..

[135] Qiu S., Sun H., Zhang A.H., Xu H.Y., Yan G.L., Han Y., Wang X.J. (2014). Natural alkaloids: Basic aspects, biological roles, and future perspectives. Chin. J. Nat. Med..

[136] Dang T.T., Onoyovwi A., Farrow S.C., Facchini P.J. (2012). Biochemical genomics for gene discovery in benzylisoquinoline alkaloid biosynthesis in opium poppy and related species. Methods Enzymol..

[137] Shan F., Yang R., Ji T., Jiao F. (2016). Vitamin C Inhibits Aggravated Eryptosis by Hydrogen Peroxide in Glucose-6-Phosphated Dehydrogenase Deficiency. Cell Physiol. Biochem..

